# The burden of periprosthetic joint infections: patient-reported outcomes and qualitative insights into periprosthetic joint infections

**DOI:** 10.5194/jbji-10-277-2025

**Published:** 2025-08-12

**Authors:** Franz-Joseph Dally, Frido Kixmöller, Frederic Bludau, Sascha Gravius, Ali Darwich, Marcel Betsch

**Affiliations:** 1 Department of Orthopaedic and Trauma Surgery, University Medical Centre Mannheim, Medical Faculty Mannheim, University of Heidelberg, Theodor-Kutzer-Ufer 1–3, 68167 Mannheim, Germany; 2 Department of Trauma and Orthopaedic Surgery, University Erlangen, Maximiliansplatz 2, 91054 Erlangen, Germany

## Abstract

**Introduction**: Periprosthetic joint infections (PJIs) have a severe physical impact and impose a significant psychological burden. This study aimed to compare patient-reported outcome measures (PROMs) and qualitative interview data within the same study cohort. **Methods**: A total of 28 PJI patients were identified after completing treatment for hip or knee PJIs. Qualitative interviews were conducted, and PROMs – such as the hip disability and osteoarthritis outcome score (HOOS), knee injury and osteoarthritis outcome score (KOOS), hospital anxiety and depression scale – total (HADS-T) score, and brief pain inventory (BPI) score – were assessed. The data were then evaluated for correlations between the PROMs and the qualitative interview findings. **Results**: A total of 20 out of 28 (71.4 %) patients scored above the accepted threshold of ≥10 on the HADS-T. A total of 8 out of 28 (28.6 %) patients scored low on the HADS-T. Through semi-structured interviews, we further evaluated the two groups: a high-HADS-T-scoring group and a low-HADS-T-scoring group. PJI patients scoring high on the HADS-T experienced a heavier psychological burden than those scoring low on the HADS-T. Our qualitative data show that the high-HADS-T-scoring group perceived their PJI experience as troubling and psychologically distressing; moreover patients in the high-HADS-T-scoring group did not deal with the PJI as well as those in the low-HADS-T-scoring group. **Conclusions**: This study provides valuable information regarding the screening of PJI patients who are at risk of psychological disorders using the HADS-T. Following screening, it also provides insight into which patients should be closely monitored and which patients should be offered professional psychological support, as the latter resource is limited and needs to be distributed sensibly. PJI patients scoring above ≥10 on the HADS-T are high-risk patients and should be offered professional psychological support.

## 1 Introduction

Periprosthetic joint infection (PJI) is a potentially life-threatening condition with a high mortality rate, up to 5 times higher than for elective joint replacement (Wildeman et al., 2021; Xu et al., 2023). It has been shown that the 10-year mortality rate for PJI patients is higher than for many malignant tumors (Thompson et al., 2022). PJI should also be regarded as a chronic illness, with some patients requiring salvage surgeries, chronic fistulas or life-long antibiotic suppressive therapy (Aggarwal et al., 2013). A German study group found that PJI patients are at the highest risk of depression just before knee reimplantation, PJI patients fear disease progression and the impact of PJI on patients' lives may be comparable to those of cancer patients (Knebel et al., 2020).

A 10-year follow-up in Sweden showed a worse quality of life and worse functional outcome for patients with PJI of the hip compared to total hip arthroplasty (THA) patients without an infection (Wildeman et al., 2021).

Qualitative studies provide deeper insights into clinical topics and allow a participant to elaborate on their experiences, attitudes and feelings in response to specific life events (Ringborg et al., 2022; Mallon et al., 2018; Tenny et al., 2023; Cleland, 2017; Moore et al., 2015; Rowland et al., 2023).

With the aim of gaining a deeper understanding of patients' experiences in relation to their patient-reported outcome measures (PROMs) data, our study correlated PROMs with qualitative interview data. We hypothesized that we would gain further insight into which patients are at the highest risk of mental disorders and would benefit the most from professional psychological support. Integrating qualitative data with PROM results, we hypothesized that the hospital anxiety and depression scale – total (HADS-T) score would primarily be impacted and supported, as qualitative data focus more on the patients' subjective experiences and feelings and less on functional outcome.

## 2 Materials and methods

From April 2022 to January 2024, a total of 28 PJI patients were interviewed. PJI patients treated at our institution had been diagnosed according to the Musculoskeletal Infection Society (MSIS) criteria (Parvizi et al., 2018). An invitation was sent to the patients via email asking them to participate in a telephone interview.

**Figure 1:**
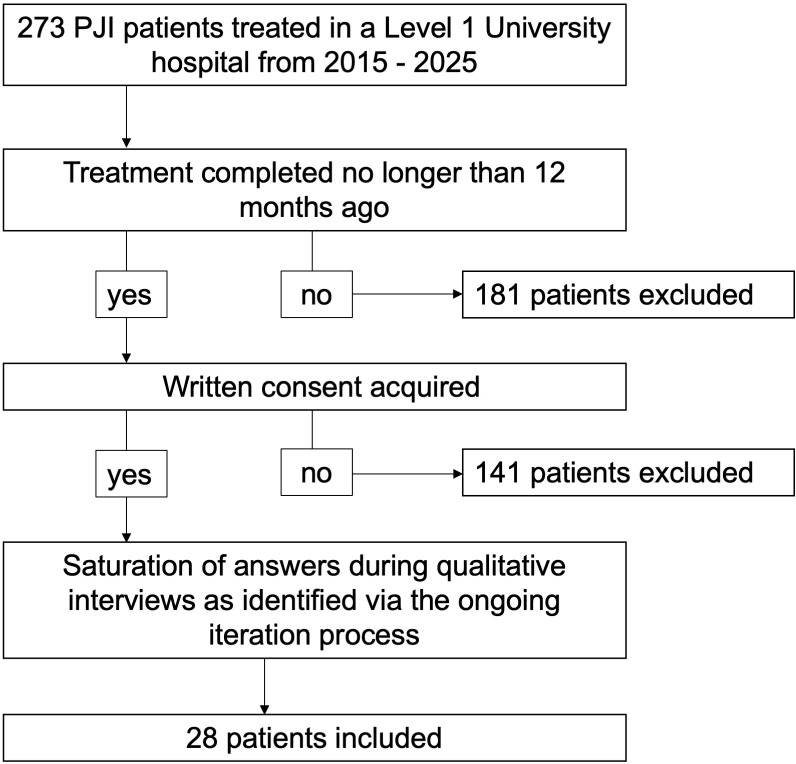
Flowchart of study inclusion.

The final study population size was determined through an ongoing iterative process (Fig. 1), as established in qualitative studies. The initial coding, categorization and theme development occurred in parallel with data collection and interviews to prevent data redundancy. The data collection and interviews ceased when theoretical saturation was reached (Braun, 2006; Clarke and Braun, 2021; Grossoehme, 2014).

Ethics approval from the local ethics board was obtained before the start of the study (approval no. 2022-531). All participating patients gave their written consent. The study protocol was developed in accordance with the Consolidated Criteria for Reporting Qualitative Research (COREQ) (Tong et al., 2007).

### 2.1 Data collection and analysis

After obtaining consent, we conducted telephone interviews with the PJI patients. Each interview lasted approximately 30–45 min, depending on the range of responses provided by the participants. We first reviewed the established and validated PROMs questionnaires.

We used the hospital anxiety and depression scale – total (HADS-T) to screen for anxiety and stress (Annunziata et al., 2020; Mitchell et al., 2010) and the brief pain inventory (BPI) scale for pain assessment and the assessment of interference with activities during daily life (Cleeland and Ryan, 1994). Functional outcome was determined via the hip disability and osteoarthritis outcome score (HOOS) and the knee injury and osteoarthritis outcome score (KOOS) (Nilsdotter et al., 2003; Roos et al., 1998).

The HADS-T is a self-report questionnaire specifically designed to screen patients in non-psychiatric settings. It has been validated for clinical use in cancer and palliative patients and is considered a robust screening tool for anxiety, depression and mixed mental disorders. The HADS-T consists of 14 questions, with 7 focused on anxiety and 7 focused on depression. Although the cutoffs for the HADS-T may vary, a score above 10 is considered high and indicates a severe risk of anxiety and depression; thus, the threshold ≥ 10 has been validated (Annunziata et al., 2020; Mitchell et al., 2010; Schellekens et al., 2016).

The BPI rapidly assesses the severity of pain and its effect on function. It has been evaluated in osteoarthritis, along with its counterpart the BPI interference scale which measures the effect on the activities of daily life (Cleeland and Ryan, 1994; Kapstad et al., 2008; Mendoza et al., 2006; Williams et al., 2006).

#### 2.1.1 Semi-structured interview

The interview questions were developed by the study team, reviewed by at least two additional authors and finalized following pilot interviews. A total of two pilot interviews were conducted to refine the questions and the structural order of the telephone interviews. All semi-structured interviews were carried out by a single trained interviewer (Frido Kixmöller). The transcripts were de-identified/pseudonymized. A method of active passivity was employed during the interview (Malterud, 2001); the interview questions are available in the Supplement. At least three team members (Franz-Joseph Dally, Frido Kixmöller and Marcel Betsch) conducted a thematic analysis of the transcripts following the inductive process described by Braun and Clarke (Braun, 2006). The authors familiarized themselves with the data by reading and rereading the transcripts. First, the data points were reviewed individually, leading to the identification of key themes (Malterud, 2001; Tenny et al., 2023; Braun, 2006). The coding process was supported by commercial software (MAXQDA software; VERBI Software GmbH, Berlin, Germany) (Vignato et al., 2022). To ensure a trustworthy process, multiple team meetings were conducted before reaching consensus on the themes. The authors collectively followed the coding phases outlined by Clarke and Braun (2021).

#### 2.1.2 Quantitative data

After receiving the participants' consent, we gathered information on the type of primary arthroplasty, the patient's body mass index (BMI), the number of surgeries, the pathogens detected and the type of infection (acute vs. chronic).

### 2.2 Statistical analysis

The questionnaire data were analyzed descriptively and are reported as means with their associated standard deviations (SDs). A t test for independent samples was performed, with the level of significance set at p<0.05 . The statistical analysis was conducted using Microsoft Excel^®^ (Microsoft Corp. Excel 2019).

### 2.3 Demographics

Demographic details are depicted in Table 1. The mean age of the patients was 68.8 years (SD ± 9 years). Of the 28 total patients, we included 14 (50 %) males and 14 (50 %) females.

**Table 1:** Key characteristics of the study population.

Parameters	All patients (28)	THA patients (12)	TKA patients (16)
	N (%)	N (%)	N (%)
Age	mean 68.8 years	mean 67 years	mean 70.1 years
	(SD ± 9 years)	(SD ± 8.9 years)	(SD ± 8.8 years)
Sex			
Male	14 (50)	6 (50)	8 (50)
Female	14 (50)	6 (50)	8 (50)
BMI	mean 29.9 kg m^−2^	mean 29.9 kg m^−2^	mean 29.6 kg m^−2^
	(SD ± 6.5 kg m^−2^)	(SD ± 6.7 kg m^−2^)	(SD ± 6.8 kg m^−2^)

THA: total hip arthroplasty; TKA: total knee arthroplasty; BMI: body mass index.

## 3 Results

### 3.1 Study population

As shown in Table 2, 11 of 28 patients (39 %) had a monomicrobial infection, 14 of 28 (50 %) developed a polymicrobial infection, and 3 of 28 (11 %) had negative cultures with no pathogen detected during treatment. A total of 17 of 28 patients (60 %) received spacer implantation at some point during their treatment. Most patients (22 of 28, 79 %) were discharged with a prosthesis or a modular revision prosthesis.

**Table 2:** Key characteristics of the study population.

Parameters N (%)	All	THA	TKA
	patients	patients	patients
	(28)	(12)	(16)
PJIs			
Acute	6 (21)	4 (33)	2 (13)
Chronic	13 (46)	4 (33)	9 (56)
Inconclusive (referred patients)	9 (32)	4 (33)	5 (31)
Primary prosthesis implanted			
TKA	16 (57)		
THA	12 (43)		
Infection type			
Monomicrobial	11 (39)	5 (42)	6 (55)
Polymicrobial	14 (50)	6 (50)	8 (50)
Negative cultures	3 (11)	1 (8)	2 (67)
Revision protocol			
DAIR	8 (29)	5 (42)	3 (19)
Multi-stage revision surgeries	20 (71)	7 (58)	13 (71)
Spacer implantations	17 (60)	5 (42)	12 (75)
Last known outcome			
Retained prosthesis or revision prosthesis	22 (79)	10 (83)	12 (75)
Girdlestone	2 (7)	2 (17)	–
Arthrodesis	3 (11)	–	3 (19)
Amputation	1 (4)	–	1 (6)

THA: total hip arthroplasty; TKA: total knee arthroplasty; DAIR: debridement, antibiotics and implant retention.

### 3.2 Patient-reported outcome measures

As depicted in Table 3, DAIR THA patients demonstrated good functional scores (HOOS), with an average of 78 % (SD ± 11). DAIR total knee arthroplasty (TKA) patients had significantly worse functional scores (KOOS), with an average of 47 % (SD ± 7; p=0.003 ). Multi-stage PJI patients of the hip ( p=0.003 ) and multistage revision PJI patients of the knee ( p=0.01 ) also showed worse outcomes in comparison to DAIR PJI patients.

**Table 3:** Results for the HOOS, KOOS, HADS, BPI pain and BPI interference measures.

Patient-reported	All patients	THA	TKA	DAIR	Multi-stage revision
outcome					surgeries
measures					
HOOS	–	56 (SD ± 21.4)	–	78 (SD ± 11) (THA)	**42** (**SD** ± **16.2**) (**THA**)
					p=0.003
KOOS	–	–	48 (SD ± 15.1)	**47** (**SD** ± **7**) (**TKA**)	**49** (**SD** ± **17.7**) (**TKA**)
				p=0.003	p=0.01
HADS-T	15.8 (SD ± 8.8)	13.5 (SD ± 7.9)	17.4 (SD ± 9.3)	16.6 (SD ± 11.0)	17.4 (SD ± 9.3)
BPI pain	3.9 (SD ± 1.2)	3.6 (SD ± 0.8)	4.0 (SD ± 1.5)	4.0 (SD ± 1.5)	3.9 (SD ± 1.5)
BPI interference	5.3 (SD ± 1.9)	4.2 (SD ± 1.5)	**6.4** (**SD** ± **1,7**)	5.3 (SD ± 2)	5.0 (SD ± 2.5)
with daily activities			p=0.02		

HOOS: hip disability and osteoarthritis outcome score; KOOS: knee injury and osteoarthritis outcome score; HADS-T: hospital anxiety and depression scale – total; BPI: brief pain inventory; THA: total hip arthroplasty; TKA: total knee arthroplasty; DAIR: debridement antibiotics and implant retention. Bold font was used for significant findings with p<0.05 .

All patients scored high on the hospital anxiety and depression scale (HADS), with TKA patients scoring higher than THA patients (17 vs. 13 points, SD ± 9.3 vs. 7.5; p=0.11 ). Looking closely at the HADS-T data, only 8 of 28 patients (28.6 %) had a score below the validated cutoff point ( ≥10 ), meaning that 20 of 28 (71.4) PJI patients in our study cohort (who scored above that cutoff) were at high risk of anxiety and depression. On the BPI interference scale, PJI patients of the knee were significantly more negatively impacted in their daily life than PJI patients of the hip (6.4 SD ± 1.7 vs. 4.2 SD ± 1.5; p=0.02 ). Further results are depicted in Table 3.

A total of 51 different pathogens were detected, with *Staphylococcus epidermidis* being the most prominent, found in 14 of 51 cases (27 %). A detailed table is available in the Supplement.

### 3.3 Qualitative results

**Figure 2:**
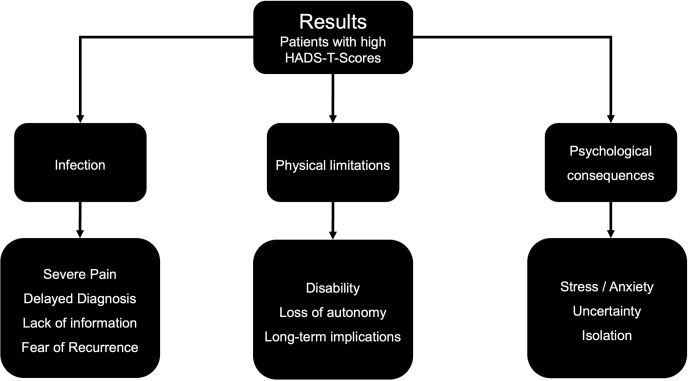
Main themes and subthemes for PJI patients with high HADS-T scores.

**Figure 3:**
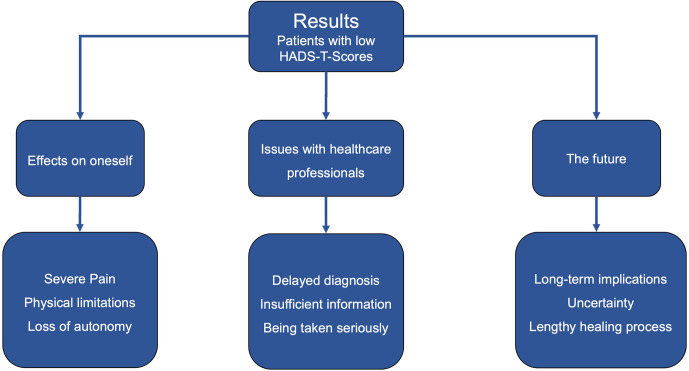
Main themes and subthemes for PJI patients with low HADS-T scores.

We recognized differences in perceived patient experiences by separating them into groups based on their HADS-T scores: those scoring high ( ≥10 ) and those scoring below the HADS-T cutoff ( <10 ). After a deductive, multistep analysis, three main themes that reflected the biographical disturbances and disruptions experienced for each (low-HADS-T and high-HADS-T) group were identified (Figs. 2 and 3).

The primary difference between PJI patients with high HADS-T scores and those with low HADS-T scores was the absence of psychological issues in the interview data of the low-HADS-T-scoring group. In part, similar themes and subthemes emerged for both groups, but how PJI patients responded, framed and weighted their answers contributed to the differences in grouping, coding, and theme development. This explains the contrasting distribution of importance and difference in perception (regarding the same topic) between the two PJI groups.

For the high-HADS-T-scoring group uncertainty caused emotional stress and significant anxiety, as opposed to the low-HADS-T-scoring patients. High-HADS-T-scoring patients reported that the uncertainty surrounding the entire process of fighting the PJI took a substantial physical and emotional toll. The experience of uncertainty was perceived in a perilous manner by the high-HADS-T-scoring group. Additionally, challenges during therapy led to friends and family distancing themselves, leaving the PJI patients feeling isolated and alone. While low-HADS-T-scoring patients seemed to find ways to manage these situations, the high-HADS-T-scoring group felt overwhelmed and depressed. We observed that, fundamentally, PJI patients with high HADS-T scores were suffering on a psychological level, with notable psychological distress and disturbances. Some appeared to be at risk of developing mental disorders, such as anxiety and depression. Exemplary quotes illustrating these themes are presented in the Supplement.

For the low-HADS-T-scoring group, we identified three main themes, supported by subthemes (Fig. 3). The first theme was the effects that the PJI had on the patients themselves. Patients reported that they were experiencing severe pain, while others also had trouble dealing with the physical limitations which, in turn, caused issues due to a loss of autonomy. The second main theme was issues with healthcare professionals. Patients suffered due to their diagnosis being delayed; moreover, they felt that they had been given insufficient information during their treatment and during the initial total joint arthroplasty, especially with respect to PJI. In some instances, patients felt that they were not being taken seriously. The third main theme was issues and implications regarding their future: some patients were fearful of long-term implications, some struggled with a general sense of uncertainty and some noted that they were worried about whether healing might be possible at all. Most notably, however, the low-HADS-scoring patients reported no psychological issues; therefore, psychological distress did not become a main or subtheme. Exemplary quotes are presented in the Supplement.

## 4 Discussion

The findings in our study demonstrate how personal, subjective and wide-ranging the experiences of PJI patients are as well as how deeply impacted patients can be. The physical, psychological and social burdens caused by PJI are significant. It is widely agreed that swift diagnosis, followed by thorough, often surgical, treatment and a multifaceted anti-infective therapy, is crucial when facing PJI (McNally et al., 2021; Parvizi et al., 2018; Darwich et al., 2021).

For the first time, we compared PROMs within the same study cohort alongside qualitative interview data. Our study showed that PJI patients are concerned about their future, the progression and recurrence of infection, their dependence on external help, and the long-term implications that PJI could have on their lives. These findings are in line with those of a German study group (Knebel et al., 2020).

The same study group found that PJI patients are at the highest risk of depression just before replantation, as assessed using the Patient Health Questionnaire (PHQ-4) (Knebel et al., 2020). Furthermore, a significant number of PJI patients may develop psychological issues and have a serious fear of disease progression, and this may have an impact on their lives that is comparable to the effects experienced by cancer patients (Knebel et al., 2020). To assess this, the study group used the PA-F-KF questionnaire, a short form of the progressive anxiety form that has been validated for cancer patients (Knebel et al., 2020).

Our study showed that the HADS-T is effective with respect to screening for psychological distress in PJI patients and that it can also successfully identify those without psychological issues.

More than two-thirds of all PJI patients scored above the validated cutoff point for anxiety and depression according to the HADS-T, which has been thoroughly evaluated in cancer patients and other patient groups besides PJI patients and is considered a robust screening tool (Annunziata et al., 2020; Mitchell et al., 2010). We grouped our PJI cohort into high-HADS-T-scoring and low-HADS-T-scoring groups based on the aforementioned cutoff ( ≥10 ) (Annunziata et al., 2020; Mitchell et al., 2010; Schellekens et al., 2016). By correlating these results with our semi-structured qualitative data, we were able to examine the cutoff for PJI patients. In the interview data from the high-HADS-T-scoring patients, psychological distress, anxiety and depression emerged as prominent and significant themes. These findings align with a recently published meta-analysis that validated the HADS-T as a screening tool, demonstrating high sensitivity when cases were evaluated through semi-structured psychiatric interviews (Mitchell et al., 2010).

It was very noticeable that, while low-HADS-T-scoring patients also experienced uncertainty, they did not perceive it as psychologically distressing and generally appeared to handle issues differently. This led to the same subtheme being grouped under a different main theme, showcasing the strength of qualitative research. Distinctions within responses regarding a single topic can be interpreted only through a thematic and deductive analytical approach unique to qualitative research (Braun, 2006; Clarke and Braun, 2021). As Singer et al. (2009) pointed out, whether or not a patient who scores highly on the HADS-T requires psychosocial support depends on comorbidities, the level of social support and the patient's desire for help. Our research and general knowledge about PJI support the points made by the aforementioned authors (Singer et al., 2009), who stated that (1) PJI is a serious condition which can be considered to be comorbid with mental disorders and (2) social support is often strained during PJI treatment, with many PJI patients requiring support on many levels (for instance, physical and emotional). Our study further demonstrates that PJI patients with high HADS-T scores exhibit significant psychological distress and disturbances. By correlating the high-HADS-T-scoring patients with their qualitative interview results, we were able to validate the screening strength of the HADS-T for PJI patients. These patients explicitly reported feeling anxious and depressed when discussing the impact of PJI. Considering both the findings of Singer et al. (2009) and our study results, we conclude that a PJI patient who scores highly on the HADS-T is at high risk of psychological distress and mental disorders and, thus, warrants professional psychological consultation.

However, there are some limitations that need to be addressed. One limitation is the singularity of time points for data collection. It might have been beneficial to collect PROMs data at various stages of the patients' treatment course and conduct interviews concurrently. This approach could have allowed for the examination of whether PROMs and qualitative interview results (themes and main themes) differ over time and might have given a better understanding of the specific impacts on a PJI patient at various treatment steps (e.g., after explantation or before replantation). Similarly, collecting the HADS-T data at different points during the PJI patient's therapy could provide insights into when psychological support or professional consultation is most needed. Another limitation is the choice of the HADS-T cutoff. The literature presents a range of cutoffs (Singer et al., 2009). We followed the cutoff with acceptable pooled results, as recommended by a recent meta-analysis (Mitchell et al., 2010).

## 5 Conclusion

In conclusion, our study shows that integrating qualitative interviews with PROMs, particularly the HADS-T, enhances our understanding of the multifaceted burden of PJI. High HADS-T scores ( ≥10 ) reliably identify patients experiencing significant psychological distress, underscoring the need for early, targeted psychosocial interventions. Despite limitations regarding data collection timing and cutoff variability, our integrated approach emphasizes the value of combining quantitative and qualitative methods to improve patient care during PJI treatment.

## Supplement

10.5194/jbji-10-277-2025-supplement The supplement related to this article is available online at https://doi.org/10.5194/jbji-10-277-2025-supplement.

## Supplement

10.5194/jbji-10-277-2025-supplement
10.5194/jbji-10-277-2025-supplement The supplement related to this article is available online at https://doi.org/10.5194/jbji-10-277-2025-supplement.

## Data Availability

The data presented in this study are available upon request from the corresponding author.

## Appendix

The supplement related to this article is available online at https://doi.org/10.5194/jbji-10-277-2025-supplement.
