# Supplementary material for: The burden of periprosthetic joint infections: patient-reported outcomes and qualitative insights into periprosthetic joint infections
Source: J Bone Jt Infect. 2025 Aug 12;10(4):277–84. doi: 10.5194/jbji-10-277-2025 (PMC12356111; doi:10.5194/jbji-10-277-2025)
Supplement: The supplement related to this article is available online at https://doi.org/10.5194/jbji-10-277-2025-supplement. [file jbji-10-277-2025-supplement.pdf]

Supplement of J. Bone Joint Infect., 10, 277–284, 2025
https://doi.org/10.5194/jbji-10-277-2025-supplement
© Author(s) 2025. CC BY 4.0 License.

Journal of Bone and Joint Infection
Open Access

Supplement of

The burden of periprosthetic joint infections: patient-reported outcomes and qualitative insights into periprosthetic joint infections

Franz-Joseph Dally et al.

Correspondence to: Franz-Joseph Dally (franz.dally@umm.de)

The copyright of individual parts of the supplement might differ from the article licence.

Supplement
Questions for the qualitative interview:

1) The onset of the infection
- How did the infection initially affect you?
- Explain your feelings about the reactions and perceptions of those around you, for example, whether your infection was taken seriously from the start.
- Describe your impression of whether you felt that your infection was diagnosed early and appropriate steps were taken?
2) The process of diagnosis and the experience of subsequent treatment
- What type of treatment was initiated after the diagnosis was made? (Surgery and/or antibiotic treatment/physiotherapy etc.)
- How did you feel about the process from the first appearance of symptoms to the actual
diagnosis?
- Describe your trust in the upcoming treatment. What expectations did you have about the outcome/course of the illness?
- Explain whether you felt insecure and unclear about your own illness? If yes, please describe how the feeling of insecurity was triggered and how the insecurity affected you or still affects you? - To what extent did you receive support from those around you at that time? - How stressful was the diagnosis and the subsequent treatment for you? - Did you feel that you had been informed about all the steps in the diagnosis and treatment? How would you generally describe the communication during the treatment?
3) Physical limitations and problems since the infection
- Please explain what limitations and problems the infection has caused you from a physical perspective?
- Compare your physical performance before the infection with your condition after the infection. Where do you feel limitations? Do you notice limitations in everyday activities and if so, please explain these limitations.
- Do you feel the infections are a long-term physical health limitation for your life? What physical limitations do you specifically have?
4) Mental limitations and problems since the infection
- Please explain what limitations and problems the infection has caused you from a mental and emotional perspective.
- How has your mental and emotional well-being been in general since the infection?
- In what way has the infection affected personal relationships? (for example with your partner and friends)
- Please describe your emotional state since the infection. Have there been any changes in your emotional state since then? (e.g. are some feelings more present and/or worse than before?)
- Have you had or still have feelings of isolation/loneliness since the infection?
- Do you feel that your quality of life has been reduced since the infection?
- What significance did/does the pain of the infection have for your psychological well-being?
- In relation to your infection, which symptoms did you find particularly stressful?
5) Long-term consequences of the periprosthetic infection
- Please name the most important aspects in your life that have influenced your life since the infection. What changes in your life has the infection led to?

- What limitations do you still experience today as a result of the infection you have had?
- In what way has the infection changed your relationship with those around you in the long term? Were social contacts affected by the infection? Have you felt a change in social support since the infection?
- When you think about yourself before the infection, what is the biggest difference to you now?
- What experiences would you tell a patient recently diagnosed with periprosthetic infection about? When you think about your experiences with the infection – what would you tell a patient recently diagnosed with periprosthetic infection? What would be most important for the patient to know in advance? -- Describe how your trust in the treating doctors/clinic staff has generally changed between the time of diagnosis and now.

Table S1:
Pathogens detected

**Pathogens = 51**	**N (%)**	**One-stage revision pathogens (DAIR) N (%)**	**Multi-stage revision (MSR) pathogens N (%)**	
Staphylococcus Epidermidis	*14 (27)*	*7 / 8 (88) DAIR were mono-microbial N = 8*	*4 / 20 MSR (20) mono-microbial N = 4*	*14 / 20 (70) poly-microbial N = 39*
Staphylococcus Aureus	*11 (22)*	*Staphylococcus Aureus 4 (57)*	4 / 4 (100) Staphylococcus Epidermidis	*Staphylococcus Epidermidis 13 (33)*
Staphylococcus Capitis	*4 (8)*	*Staphylococcus Epidermidis 1 (14)*		*Staphylococcus Aureus 7 (18)*
Staphylococcus Hominis	*3 (6)*	*Pseudomonas oryzihabitans 1 (14)*		*Staphylococcus Capitis 4 (10)*
Cutibacterium Acnes	*2 (6)*	*Staphylococcus saccharolyticus 1 (14)*		*Staphylococcus Hominis 3 (8)*
Corynebacterium amycolatum	*1 (2)*			*Cutibacterium Acnes 2 (5)*
Enterococcus faecialis	*1 (2)*			*Corynebacterium amycolatum 1 (3)*
Staphylococcus Haemolyticus	*1 (2)*			*Enterococcus faecialis 1 (3)*
Staphylococcus caprae	*1 (2)*			*Staphylococcus Haemolyticus 1 (3)*
Staphylococcus saccharolyticus	*1 (2)*			*Staphylococcus caprae 1 (3)*
Finegoldia magna	*2 (4)*			*Finegoldia magna 2 (6)*
Pseudomonas oryzihabitans	*1 (2)*			*Enterobacter cloacae spp. 1 (3)*

Enterobacter cloacae spp.	*1 (2)*			*Klebsiella pneumoniae 1 (3)*
Klebsiella pneumoniae	*1 (2)*			*Corynebakterium Tuberculostaticum 2 (6)*
Corynebakterium Tuberculostaticum	*2 (4)*			*Strreptococcus agalactiae 1 (3)*
Strreptococcus agalactiae	*1 (2)*			*Staphylokokken koagulase negativ 1 (3)*
Staphylokokken koagulase negativ	*1 (2)*			*Escherischa coli 1 (3)*
Escherischa coli	*1 (2)*			*Proprionibacterium acnes 1 (3)*
Proprionibacterium acnes	*1 (2)*			*Corynebakterium striatum 1 (3)*
Corynebakterium striatum	*1 (2)*			
no pathogens identified	*3*	*no pathogens identified 1 (12)*		*no pathogens identified 2 (10)*

DAIR = Debridement Antibiotics and implant retention, MSR: multi-stage revisions

Table S2: Quotations leading to the development of themes and key themes for “HADS high scorers”

**Key Theme**	**Infection**
Themes	Quotations
Severe pain	› „…I was suffering from **severe pain** and I kept going back for further appointments, but I always received the same answer, there was (supposed to be) no issue with the prosthesis…“<br>› „…in the end **I kept taking higher and higher doses of morphin** which my general practicioner prescribed, I was completely out of it, almost laying on the couch the entire day …“<br>› „…the surgeons in the other hospital **didn't seem to believe me when I told them I was suffering** from that much pain. …”<br>› „... I was just not expecting it, after the initial surgery everything seemed fine and **then all of the sudden there was just so much pain.** …”

Delayed diagnosis	› „…**nobody took me seriously**, after multiple appointments the chief of orthopedics finally said, let's run some more tests, and then we discovered the infection. …“<br>› „…my wound just didnt heal properly, but I was sent to the rehabilitation clinic, **after another ten days they sent me to my surgeon** …”<br>› „…they just sent me back home, they didn't habe a laboratory, I think and **after the bacteria was detected, they didn't even inform me right away, in the end I developed a putrid fistula.** ..."
Lack of information	› „…I simply didn't know how severe this infection was, and **nobody really talked to me about the severity, I thought you could compare to a common cold** and then before I knew, they told me they needed to explant the prosthesis. …“<br>› „… I started to doubt myself, but then **the doctor finally told me**, something is wrong with your prosthesis, I was actually relieved. …”<br>› „… Before the initial implantation, I think somebody briefly mentioned the possibilty of an infection, **but I couldn't grasp what that meant. When it was time to make a decision, everyone seemed to pass on the responsibilites** ..."
Fear of reccurence	› „… In the back of my mind, **there will always be a slight panic**. I still go to my GP and have blood tests done every three months ..."<br>› „… They told me, **the infection could come back at any point for the rest of my life.** ..."<br>› „… When I was told I needed another surgery (which would have been almost the 50th surgery altogether), I was about to tell them, just cut off my leg, **I am too worried for the infection to return**. ..."<br>› „…**Fear is my constant companion**, and I keep having flashbacks to the time in the hospital. ..."
**Key Theme**	**Physical limitations**
Disability	› „…I live on the second floor, I never imagined this being an issue so soon in my life, **I might have to move**. ...“<br>› „… **I can barely walk, let alone walk up and down the stairs**…“<br>› „…**My leg is a lot shorter now**, I am still trying to adjust, but it causes problems with my back, my feet**.** …“<br>› „… My life has been turned upside down, **I used to be self-sufficient, now I need help all the time.** ..."<br>› „… **I need my walker to get anywhere**, I am not enjoying this. ..."
Loss of autonomy	› „…**Driving my car, I think that is absolutely unrealistic**, I don't know how I would do that, I need to be driven everywhere now…”<br>› „…I was **hoping for help, support, of any kind really, but I didn't get any** …”<br>› „… **I can't do the things I used to when I want to**, just going for a walk, I cannot manage alone, not I have to wait until somebody helps me go for a walk. ..."
Long-term implications	› „…That really scares me. I have **existential fears**. …”<br>› „…the doctors told me, they could not perform more surgeries on my leg and **I might have to live with my pain for the rest of my life**. …”<br>› „… and then I lost my job and my financial stability, **I am 61 years old and now I live on welfare**. ..."<br>› „… you have to imagine, there have been a number of surgeries on my knee, **the quality of my bone might not take another surgery**. ..."
**Key Theme**	**Psychological consequences**

Stress/Anxiety	› „…**you cannot imagine what I have been through**, I have a deeply rooted restlessness an anxiety since my surgeries. …”<br>› „…**when I close my eyes, I am set back into the situation in the hospital**, I could not move, I was suffering. …”<br>› „…for a period, I could not use stairs and **I kept having panic attacks**, in case something happened and I did not know how to get help or what to do …”<br>› „… It is not just the leg, **I am scared, I am depressed, I cannot go outside**. ..."
Uncertainty	› „…Every time before a surgery there was this **hope of everything turning out well this time**, only to find out that there was another infection. We always had this hope and **were disappointed** after a while. …”<br>› „…The issue is that as a family you have this expectation and hope that from receiving another prosthesis everything will turn to the better and that the quality of life will improve. **None of us could foresee that this would be the result**. …”<br>› „…You just **feel exposed**, because you yourself can’t do anything, you have to trust the doctors and end up feeling helpless. …”
Isolation	› „…**my friends just left me**, they became desinterested, they did not reach out to me, that felt horrible. …“<br>› „…**I ended up having no more friends** because I couldn't interact or socialize with them anymore. …“<br>› „…I do what I can at home, **but I am completely alone**, I have no one. …“<br>› „…because I couldn't get anywhere anymore with my leg, I lost touch, **but everyone also simply stopped coming by**. ..."

Table S3: Quotations leading to the development of themes and key themes for “HADS-T low scorers”

**Key Theme**	**Effects on oneself**
Themes	Quotations
Severe pain	› „…I had **real pain, especially while I was walking**, but I had to push through…“<br>› „…when I do too much, like walk too far, or when I put too much strain on my leg, **I get swelling, effusion and pain.** …“<br>› „…there were maybe **two or three good hours there, then the pain came back** and I had to take more pain pills. …”<br>› „... I was in **severe pain, I had to cry** a lot because of it, my family was suffering from it. …”
Physical limitations	› „…**the physical limitations were imminent;** I was on crutches and a walker and had to live in a nursing home for 8 weeks. …“<br>› „…**I used to be really fit** and now I cannot walk to the store without crutches. …”<br>› „…My **leg just gets so tired, I need to take breaks** a lot, I can’t really walk**.** ..."
Loss of autonomy	› „… I **couldn’t use the toilet, couldn’t take a shower on my own**, those were severe limitations for somebody who is used to have complete autonomy. …“<br>› „… I am just so severely limited, **I can’t go grocery shopping or to the store**, somebody has to do all that. …”<br>› „… for me the worst time, was in the hospital, **always needing help for every little thing**, going to the toilet, taking a shower, body hygiene, ..."

**Key Theme**	**Issues with healthcare professionals**
Delayed diagnosis	› „…I am not a doctor, but **I felt that it took forever** until someone reacted to the samples taken ...“<br>› „… **I finally found a doctor who told me, well this is not normal, we need to run more tests**…“<br>› „… After a while I kept saying, **this does not feel normal**, I still cannot move. ..."
Insufficient information	› „…**my initial surgeon, he did not come and talk to me about the infection.** …”<br>› „…**looking back I have to say, I had no idea** how severe this infection could have been, removing the prosthesis, I was not aware that was an option …”<br>› „… **this was outside of my imagination, that these bacteria could cause such a harm**. ..."
Being taken seriously	› „…**I really felt nobody was listening to me**. …”<br>› „…it seemed to me they **were thinking I was acting**, I was not being taken seriously. …”<br>› „… my other doctors and maybe sometimes my family, so I thought, were telling me, come on, **don’t act like this (is hurting) again** ..."

**Key Theme**	**The future**
Long-term implications	› „…**this leaves the question, what if there has to be another surgery**. …”<br>› „…**I depend on help all the time,** at home I can kind of cope now. …”<br>› „…I can walk, but I am so unsteady on my feet, so to say, **it feels useless sometimes.** …”<br>› „… **I cannot use my car** and I do not see a near future where I will be able to, it does not seem safe. ..."
Uncertainty	› „…Every time before a surgery there was this **hope of everything turning out well this time**, only to find out that there was another infection. We always had this hope. …”<br>› „…I guess **I still have dreams**, going out to a dance, some simple things, like shopping. …”<br>› „…I am **certainly more cautious** about everything I do …”
Lenghty healing process	› „…**I couldn’t believe how long everything took,** all the iv antibiotics, the rehabilitation process. …“<br>› „…**it felt like I had to learn to walk again but my muscles were so weak**. …“<br>› „…I do what I can at home, **but I am completely alone**, I have no one. …“<br>› „…because I couldn't get anywhere anymore with my leg, I lost touch, **but everyone also simply stopped coming by**. ..."
